# Subgaleal coiling of the proximal and distal components of a ventriculoperitoneal shunt

**DOI:** 10.1186/1865-1380-5-15

**Published:** 2012-03-16

**Authors:** Brian T Kloss, David M Hart, LaLainia Secreti

**Affiliations:** 1 SUNY Upstate Medical University, Syracuse, NY; 2 University of Rochester Medical Center, Rochester, NY

## Abstract

Migration is a rare complication of venticuloperitoneal shunts and is thought to be associated with the "memory" of the plastic tubing and the windlass effect of neck flexion and extension. The purpose of this case report is to detail a very rare case of complete distal to proximal shunt migration.

## Case Report

**Figure 1:**
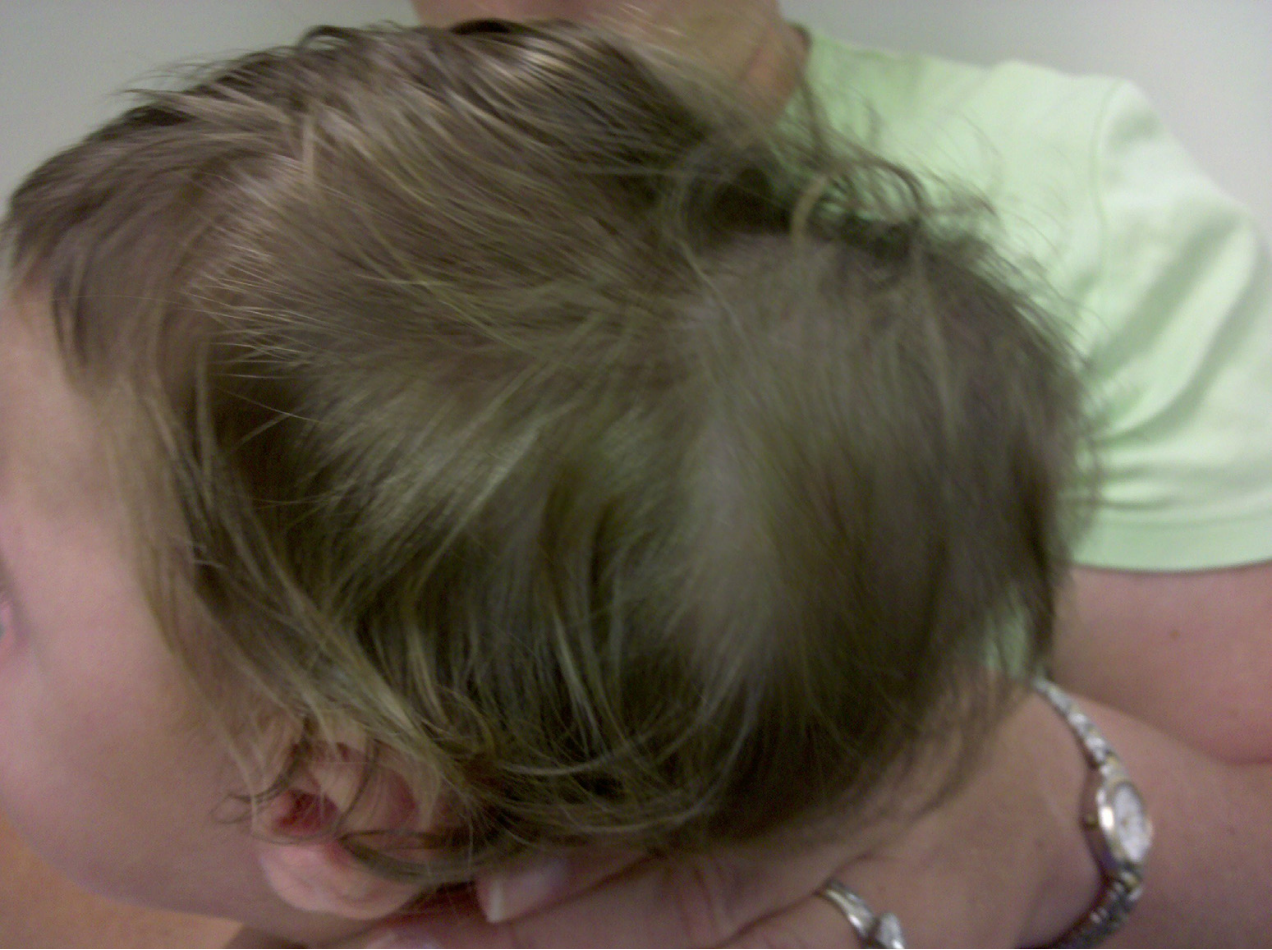
**Side Photograph Showing Coiled Shunt**.

**Figure 2:**
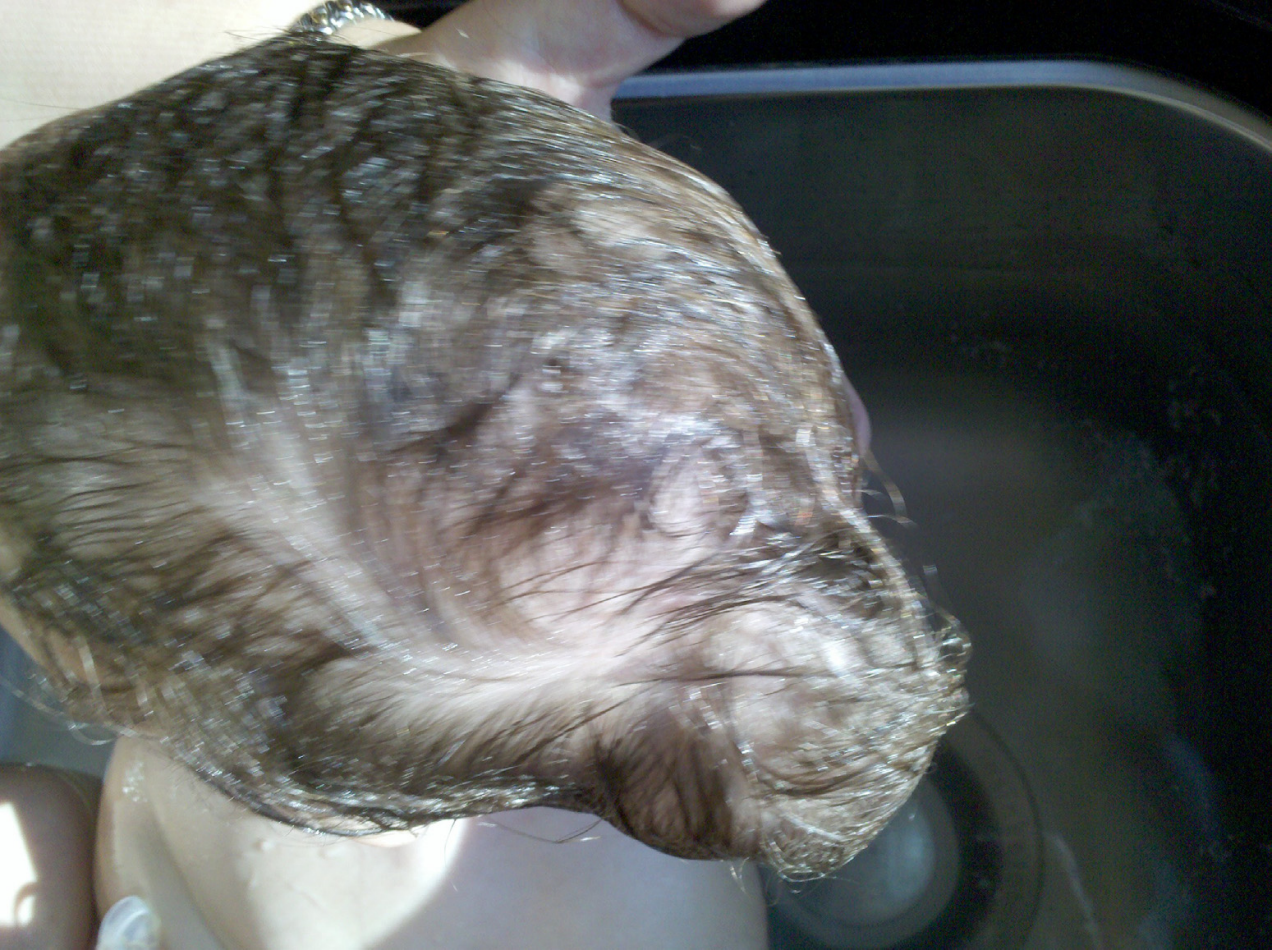
**Top Photograph Showing Coiled Shunt**.

**Figure 3:**
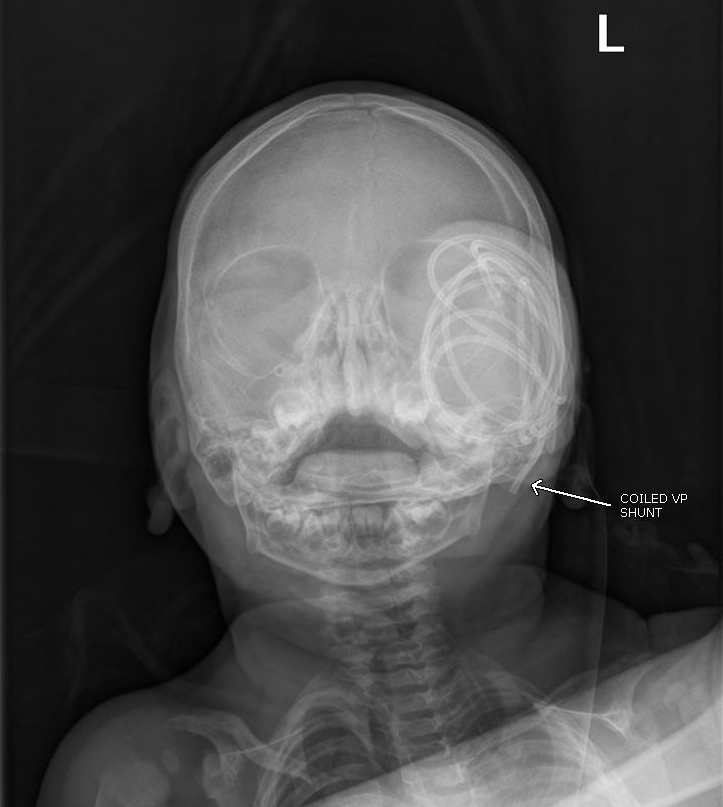
**AP Skull Showing Coiled Shunt**.

**Figure 4:**
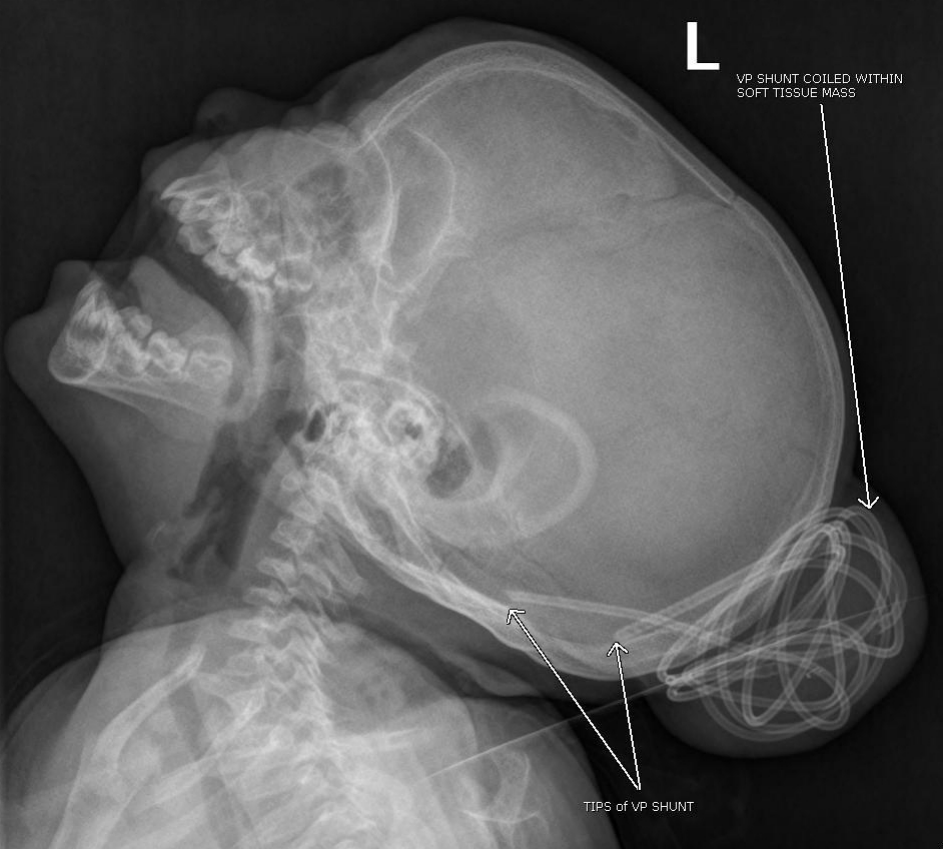
**Lateral Skull Showing Coiled Shunt**.

**Figure 5:**
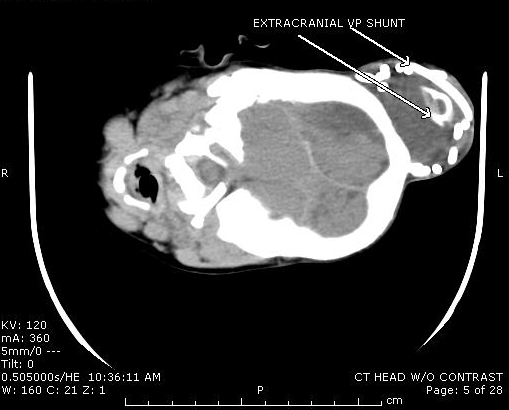
**Cross Section CT Showing Coiled Shunt**.

**Figure 6:**
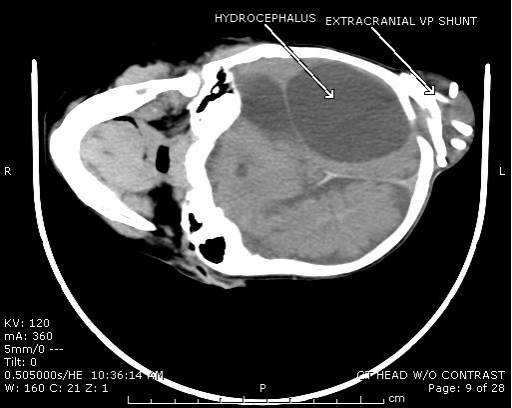
**Cross Section CT Showing Coiled Shunt and Hydrocephalus**.

An 11-month-old female with a history of multiloculated, complex intracranial cysts with shunt-dependent hydrocephalus and seizure disorder presented to the emergency department with a 2-day history of lethargy, sleepiness, nausea and vomiting. The patient was 3 days status post insertion of a left ventriculoperitoneal shunt insertion. Vital signs were stable, and physical exam revealed a large subgaleal mass at the left occipital area. (Figures 1 and 2) Skull films (Figures 3 and 4) and head CT (Figures 5 and 6) revealed that the shunt catheter was located completely outside the cranium and contained within it a 3.4 × 5.1 × 4.0-cm subgaleal fluid-filled pocket. The child was taken back to the OR, and a new shunt catheter was placed without complication. The child has since been doing well.

Significant proximal shunt migration is an extremely rare complication that has only been reported in the literature on six occasions [1-5], with the full migration of the distal and proximal aspects of the shunt catheter into a subgaleal pocket reported only twice [2,4]. The proposed mechanism for the migration of the catheter involves the "windlass" effect combined with the retained "memory" of the shunt tubing [2]. The flexion and extension of the child's neck in the days following the surgery combined with the "memory" of the plastic tubing were the likely culprits causing the shunt migration.

## Competing interests

The authors declare that they have no competing interests.

## Authors' contributions

BK oversaw and edited the case report and the organization of the images as published. DH researched the current literature and wrote the body of the paper. LS oversaw patient care in the ED and obtained verbal and written consent for publication. All authors read and approved the final manuscript.
